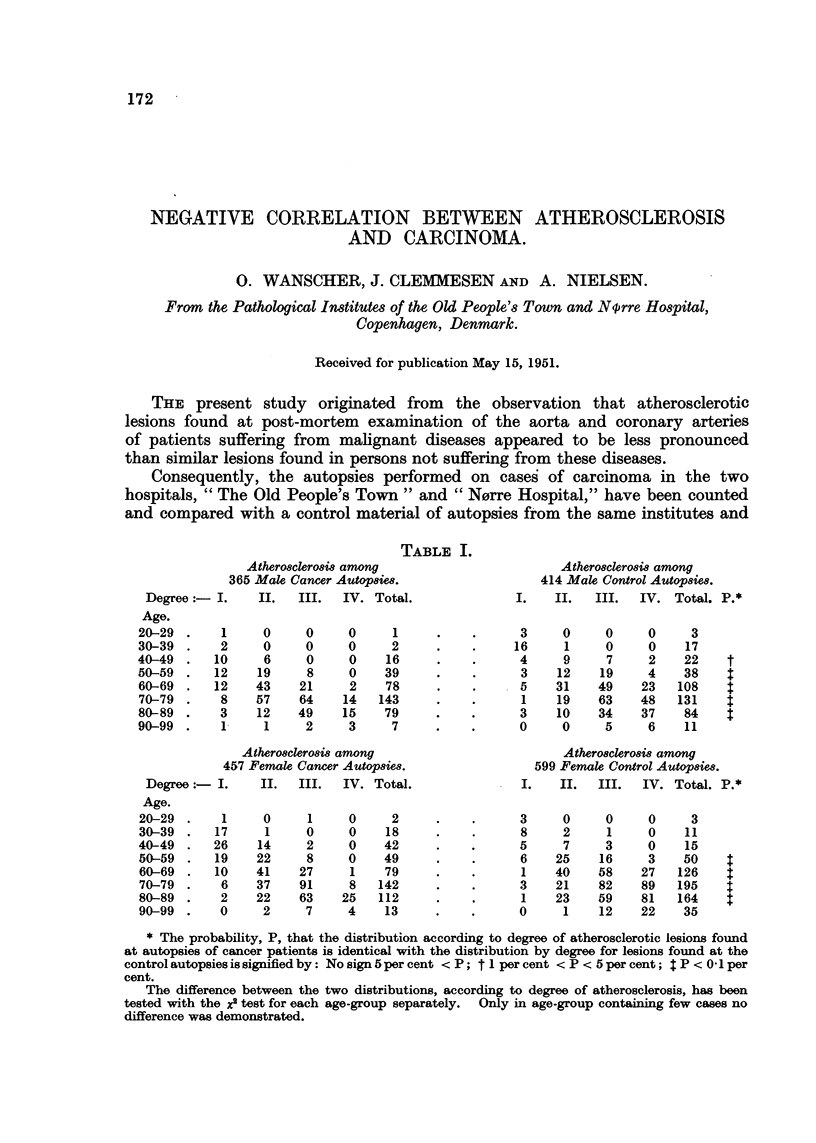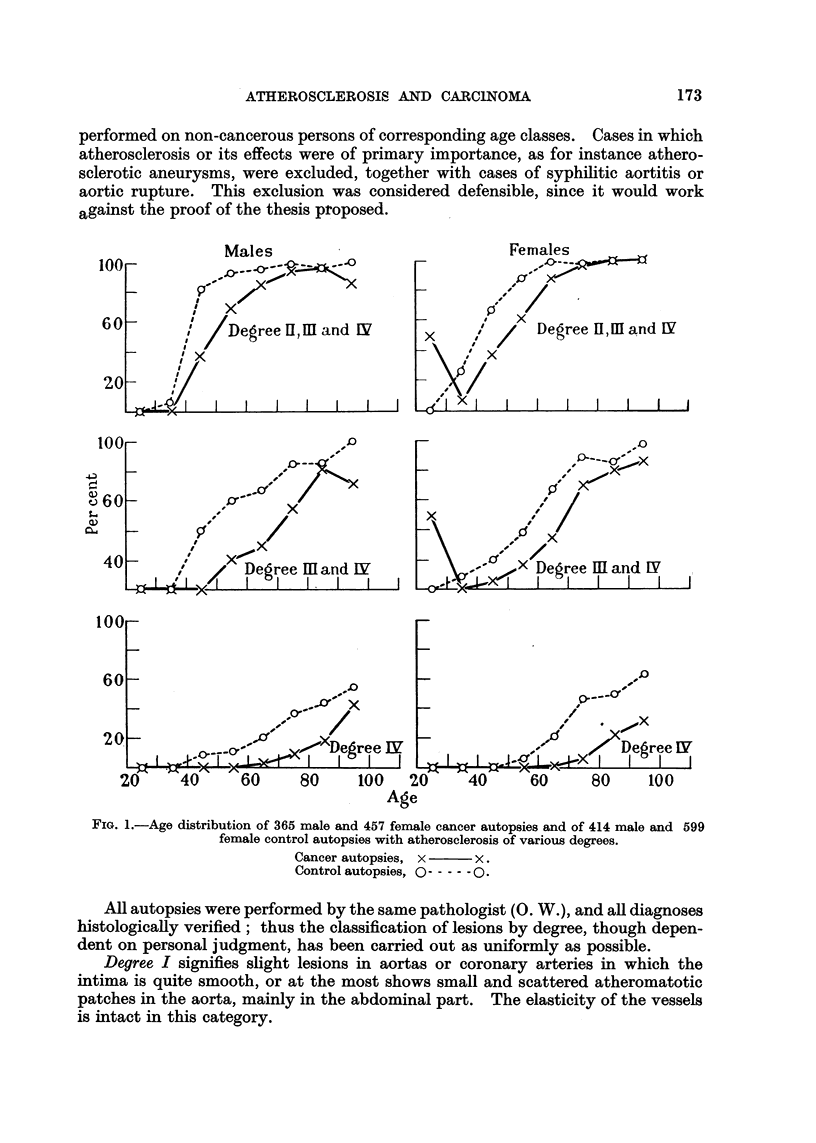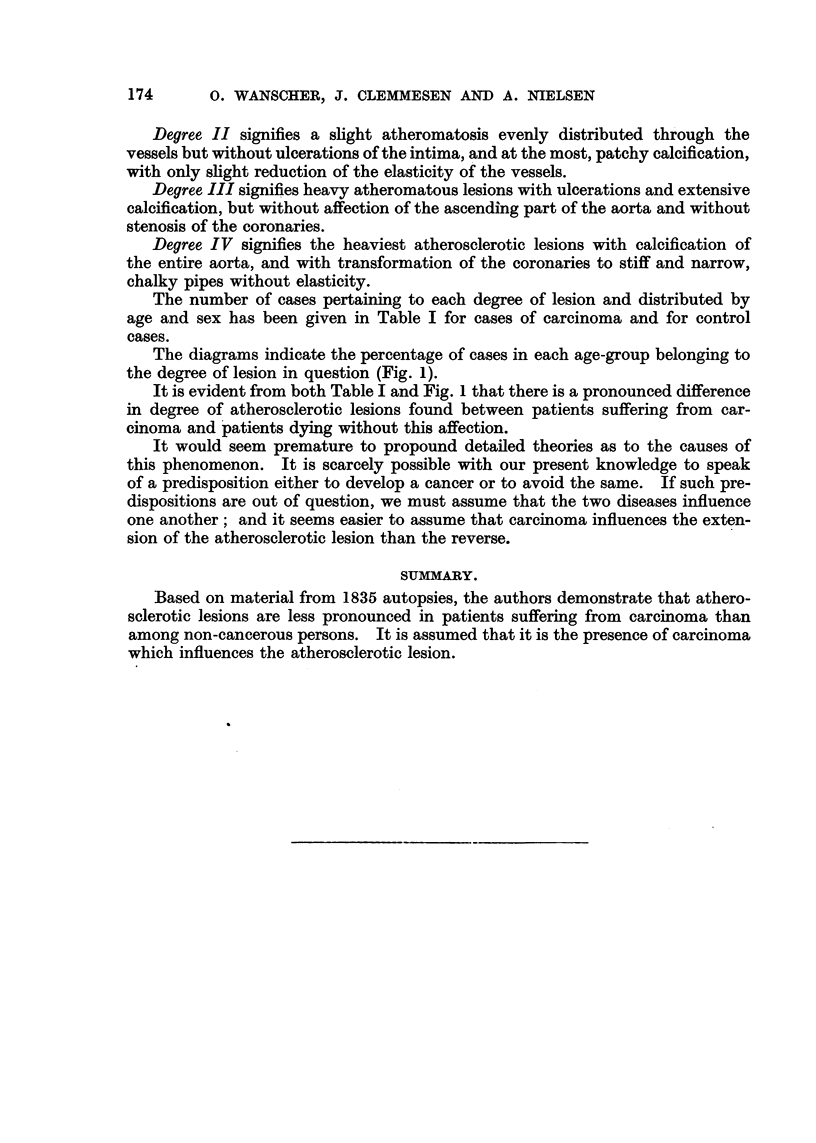# Negative Correlation Between Atherosclerosis and Carcinoma

**DOI:** 10.1038/bjc.1951.18

**Published:** 1951-06

**Authors:** O. Wanscher, J. Clemmesen, A. Nielsen


### NEGATIVE CORRELATION BETWEEN ATHEROSCLEROSIS AND CARCINOMA.

O. WANSCHER, J. CLEMMESEN AND A. NIELSEN.

*From the Pathological Institutes of the Old People's Town and Nφrre Hospital, Copenhagen, Denmark.*

Received for publication May 15, 1951.

THE present study originated from the observation that atherosclerotic lesions found at post-mortem examination of the aorta and coronary arteries of patients suffering from malignant diseases appeared to be less pronounced than similar lesions found in persons not suffering from these diseases.

Consequently, the autopsies performed on cases of carcinoma in the two hospitals, "The Old People's Town" and "Nørre Hospital," have been counted and compared with a control material of autopsies from the same institutes and

TABLE I.

| | *Atherosclerosis among 365 Male Cancer Autopsies.* | | | | | *Atherosclerosis among 414 Male Control Autopsies.* | | | | | |
|---|---|---|---|---|---|---|---|---|---|---|---|
| Degree :— | I. | II. | III. | IV. | Total. | I. | II. | III. | IV. | Total. | P.* |
| Age. | | | | | | | | | | | |
| 20–29 | 1 | 0 | 0 | 0 | 1 | 3 | 0 | 0 | 0 | 3 | |
| 30–39 | 2 | 0 | 0 | 0 | 2 | 16 | 1 | 0 | 0 | 17 | |
| 40–49 | 10 | 6 | 0 | 0 | 16 | 4 | 9 | 7 | 2 | 22 | † |
| 50–59 | 12 | 19 | 8 | 0 | 39 | 3 | 12 | 19 | 4 | 38 | ‡ |
| 60–69 | 12 | 43 | 21 | 2 | 78 | 5 | 31 | 49 | 23 | 108 | ‡ |
| 70–79 | 8 | 57 | 64 | 14 | 143 | 1 | 19 | 63 | 48 | 131 | ‡ |
| 80–89 | 3 | 12 | 49 | 15 | 79 | 3 | 10 | 34 | 37 | 84 | ‡ |
| 90–99 | 1 | 1 | 2 | 3 | 7 | 0 | 0 | 5 | 6 | 11 | |

| | *Atherosclerosis among 457 Female Cancer Autopsies.* | | | | | *Atherosclerosis among 599 Female Control Autopsies.* | | | | | |
|---|---|---|---|---|---|---|---|---|---|---|---|
| Degree :— | I. | II. | III. | IV. | Total. | I. | II. | III. | IV. | Total. | P.* |
| Age. | | | | | | | | | | | |
| 20–29 | 1 | 0 | 1 | 0 | 2 | 3 | 0 | 0 | 0 | 3 | |
| 30–39 | 17 | 1 | 0 | 0 | 18 | 8 | 2 | 1 | 0 | 11 | |
| 40–49 | 26 | 14 | 2 | 0 | 42 | 5 | 7 | 3 | 0 | 15 | |
| 50–59 | 19 | 22 | 8 | 0 | 49 | 6 | 25 | 16 | 3 | 50 | ‡ |
| 60–69 | 10 | 41 | 27 | 1 | 79 | 1 | 40 | 58 | 27 | 126 | ‡ |
| 70–79 | 6 | 37 | 91 | 8 | 142 | 3 | 21 | 82 | 89 | 195 | ‡ |
| 80–89 | 2 | 22 | 63 | 25 | 112 | 1 | 23 | 59 | 81 | 164 | ‡ |
| 90–99 | 0 | 2 | 7 | 4 | 13 | 0 | 1 | 12 | 22 | 35 | |

* The probability, P, that the distribution according to degree of atherosclerotic lesions found at autopsies of cancer patients is identical with the distribution by degree for lesions found at the control autopsies is signified by: No sign 5 per cent $< P$; † 1 per cent $< P <$ 5 per cent; ‡ $P < 0{\cdot}1$ per cent.

The difference between the two distributions, according to degree of atherosclerosis, has been tested with the $\chi^2$ test for each age-group separately. Only in age-group containing few cases no difference was demonstrated.

performed on non-cancerous persons of corresponding age classes. Cases in which atherosclerosis or its effects were of primary importance, as for instance atherosclerotic aneurysms, were excluded, together with cases of syphilitic aortitis or aortic rupture. This exclusion was considered defensible, since it would work against the proof of the thesis proposed.

FIG. 1.—Age distribution of 365 male and 457 female cancer autopsies and of 414 male and 599 female control autopsies with atherosclerosis of various degrees.

Cancer autopsies, ×———×.

Control autopsies, ○- - - - -○.

All autopsies were performed by the same pathologist (O. W.), and all diagnoses histologically verified ; thus the classification of lesions by degree, though dependent on personal judgment, has been carried out as uniformly as possible.

*Degree I* signifies slight lesions in aortas or coronary arteries in which the intima is quite smooth, or at the most shows small and scattered atheromatotic patches in the aorta, mainly in the abdominal part. The elasticity of the vessels is intact in this category.

*Degree II* signifies a slight atheromatosis evenly distributed through the vessels but without ulcerations of the intima, and at the most, patchy calcification, with only slight reduction of the elasticity of the vessels.

*Degree III* signifies heavy atheromatous lesions with ulcerations and extensive calcification, but without affection of the ascending part of the aorta and without stenosis of the coronaries.

*Degree IV* signifies the heaviest atherosclerotic lesions with calcification of the entire aorta, and with transformation of the coronaries to stiff and narrow, chalky pipes without elasticity.

The number of cases pertaining to each degree of lesion and distributed by age and sex has been given in Table I for cases of carcinoma and for control cases.

The diagrams indicate the percentage of cases in each age-group belonging to the degree of lesion in question (Fig. 1).

It is evident from both Table I and Fig. 1 that there is a pronounced difference in degree of atherosclerotic lesions found between patients suffering from carcinoma and patients dying without this affection.

It would seem premature to propound detailed theories as to the causes of this phenomenon. It is scarcely possible with our present knowledge to speak of a predisposition either to develop a cancer or to avoid the same. If such predispositions are out of question, we must assume that the two diseases influence one another ; and it seems easier to assume that carcinoma influences the extension of the atherosclerotic lesion than the reverse.

SUMMARY.

Based on material from 1835 autopsies, the authors demonstrate that atherosclerotic lesions are less pronounced in patients suffering from carcinoma than among non-cancerous persons. It is assumed that it is the presence of carcinoma which influences the atherosclerotic lesion.